# OD38 Real-World Analysis Of First-Line Maintenance Treatment For Patients With Nonsquamous Advanced/Metastatic Non-Small-Cell Lung Cancer

**DOI:** 10.1017/S0266462324001673

**Published:** 2025-01-07

**Authors:** Umit Tapan, Kelly F. Bell, Amine Aziez, Jiaqian Sun, Mandy Du, Ella Xiaoyan Du, Qi Hua, Hongbo Yang, Manasee Shah

## Abstract

**Introduction:**

Pemetrexed and immunotherapies (e.g., pembrolizumab) are approved for first-line maintenance (1LM) treatment of nonsquamous advanced/metastatic non-small-cell lung cancer (NSCLC), but real-world data on their use are limited. The objective of this study was to assess 1LM clinical outcomes, safety, and treatment patterns of immunotherapy versus immunotherapy+pemetrexed among patients with advanced/metastatic NSCLC from the EU4 (France, Germany, Italy, Spain)+UK.

**Methods:**

Data from patients in the US, Canada, and EU4+UK with nonsquamous advanced/metastatic NSCLC without targetable mutations were collected via electronic case report form. Physician-identified patients (≥18 y) in the EU4+UK were eligible for this subgroup analysis if they achieved stable disease or complete or partial response with first-line platinum-based chemotherapy+immunotherapy (January 2019 to March 2021) and received 1LM immunotherapy or immunotherapy+pemetrexed. Patients were followed from index (1LM initiation) until last physician contact or death. Outcomes were overall survival (OS), progression-free survival (PFS), treatment patterns and duration, and adverse events.

**Results:**

Among the selected 367 patients (male, 71.9%; mean±StDev age, 63.4±7.2 y; current/former smokers, 85.8%), 203 (55.3%) received immunotherapies, most commonly pembrolizumab (n=173; 85.2%), and 164 (44.7%) received immunotherapy+pemetrexed. Patients receiving immunotherapy had longer median adjusted OS and PFS compared to those receiving immunotherapy+pemetrexed (OS hazard ratio [HR]: 0.63; 95% confidence interval [CI]: 0.36, 0.90; PFS HR: 0.58; 95% CI: 0.38, 0.79). Patients receiving immunotherapy versus patients receiving immunotherapy+pemetrexed had longer median treatment duration (14.0 vs 10.3 mo; p<0.001) and were less likely to experience anemia (19.7% vs 33.5%; p<0.01). Results were similar in the overall study population.

**Conclusions:**

In this real-world study, among the selected patients with nonsquamous advanced/metastatic NSCLC who achieved stable disease or complete or partial response with first-line therapy, the addition of pemetrexed to immunotherapy in 1LM did not appear to confer a clinical benefit. Identifying treatments that can improve clinical outcomes for these patients remains an area of unmet need.

